# Supplementary material for: Phosphorylation of Arabidopsis UVR8 photoreceptor modulates protein interactions and responses to UV-B radiation
Source: Nat Commun. 2024 Feb 9;15:1221. doi: 10.1038/s41467-024-45575-7 (PMC10858049; doi:10.1038/s41467-024-45575-7)
Supplement: Supplementary file 1 — Supplementary information [file 41467_2024_45575_MOESM1_ESM.pdf]

Supplementary Information for:

Phosphorylation of Arabidopsis UVR8 photoreceptor modulates protein interactions and responses to UV-B radiation

Wei Liu, Giovanni Giuriani, Anezka Havlikova, Dezhi Li, Douglas J. Lamont, Susanne Neugart, Christos N. Velanis, Jan Petersen, Ute Hoecker, John M. Christie and Gareth I. Jenkins

Supplementary Table 1 | Example of calculation of relative ^{32}P incorporation into GFP-UVR8.

UV-B	Scintillation counting of 1 ul protein extract (cpm)	Total volume of protein extract (ul)	Total radioactivity taken into tissue (cpm)	Band intensity of western blot	Band intensity of autoradiograph	Relative ^{32}P incorporation into GFP-UVR8
**0 h**	331514	264	87519696	29298.75	19524.97	1
**24 h**	339100	236	80027600	36300.73	31925.84	1.44

Seedlings were exposed to UV-B for the indicated times and used to make a protein extract. The total radioactivity taken into the tissue was measured by scintillation counting of 1 µl of the extract of known volume. A western blot of immunoprecipitated GFP-UVR8 was subjected to autoradiography and then incubated with an appropriate antibody. Band intensities were determined using ImageJ. Relative incorporation of ^{32}P into GFP-UVR8 was adjusted for the level of radioactivity taken into the tissue and amount of GFP-UVR8 present on the blot.

Supplementary Table 2 | Mass spectrometry analysis of UVR8 phosphopeptides.

Mascot Score	Peptide
**WL Replicate 1**	
135	K.IIEALSVDGASGQHIESSNIDPSSGK.S
64	K.IIEALSVDGASGQHIESSNIDPSSGK.S
47	K.IIEALSVDGASGQHIESSNIDPSSGKSWVSPAER.Y
41	R.LGHGNSSDLFTPLPIK.A
38	R.NQNGQLGLGDTEDSLVPQKIQAFEGIR.I
33	K.SWVSPAER.Y
31	R.YAVVPDETGLTDGSSKGNGGDISVPQTDVKR.V
28	R.YAVVPDETGLTDGSSKGNGGDISVPQTDVK.R
28	R.YAVVPDETGLTDGSSKGNGGDISVPQTDVKR.V
28	K.IIEALSVDGASGQHIESSNIDPSSGKSWVSPAER.Y
25	R.LGHGNSSDLFTPLPIKALHGIR.I
23	K.FGQVGVGNNLDQCSPVQVR.F
23	R.NQNGQLGLGDTEDSLVPQKIQAFEGIR.I
**WL Replicate 2**	
60	K.IIEALSVDGASGQHIESSNIDPSSGK.S
50	R.LGHGNSSDLFTPLPIK.A
49	K.IIEALSVDGASGQHIESSNIDPSSGKSWVSPAER.Y
39	K.SWVSPAER.Y
38	R.YAVVPDETGLTDGSSKGNGGDISVPQTDVK.R
36	R.LGHGNSSDLFTPLPIKALHGIR.I
27	R.NQNGQLGLGDTEDSLVPQKIQAFEGIR.I
26	R.NQNGQLGLGDTEDSLVPQKIQAFEGIR.I
**WL Replicate 3**	
119	K.IIEALSVDGASGQHIESSNIDPSSGK.S
76	K.IIEALSVDGASGQHIESSNIDPSSGK.S
46	R.YAVVPDETGLTDGSSKGNGGDISVPQTDVK.R
44	R.LGHGNSSDLFTPLPIK.A
36	R.LGHGNSSDLFTPLPIK.A
33	K.SWVSPAER.Y
27	R.NQNGQLGLGDTEDSLVPQKIQAFEGIR.I
26	K.IIEALSVDGASGQHIESSNIDPSSGKSWVSPAER.Y

Table continued next page: UV-B replicates

UV-B Replicate 1

121	K.IIEALSVDGA**S**GQHIESSNIDPSSGK.S
44	R.LGHGNSSDLF**T**PLPIK.A
41	K.SWV**S**PAER.Y
41	R.LGHGNSSDLFTPLPIK.A
36	K.IIEALSVDGASGQHIESSNIDPSSGKSWVSPAER.Y
30	K.MVAAGAEHTAAVTEDGDLYGWGWGR.Y
25	R.YAVVPDETGLTDGSSKGNGGDISVPQTDVK.R
24	R.NQNGQLGLGDTEDSLVPQKIQAFEGIR.I
24	R.NQNGQLGLGDTEDSLVPQKIQAFEGIR.I
24	R.YAVVPDETGLTDGSSKGNGGDISVPQTDVK.R
22	R.LGHGNSSDLFTPLPIKALHGIR.I

UV-B Replicate 2

33	R.LGHGNSSDLFTPLPIKALHGIR.I
31	K.SWVSPAER.Y
27	K.IIEALSVDGASGQHIESSNIDPSSGKSWVSPAER.Y
22	K.IIEALSVDGASGQHIESSNIDPSSGKSWV**S**PAER.Y
22	R.HTMALTSDGKLYGWGWNKFGQVGVGNNLDQCSPVQVR.F

UV-B Replicate 3

41	K.SWV**S**PAER.Y
27	R.YAVVPDETGLTDGSSKGNGGDISVPQTDVK.R
26	K.IIEALSVDGASGQHIESSNIDPSSGKSWVSPAER.Y
24	K.QIACGDSHCLAVTMEGEVQSWGR.N
24	R.NQNGQLGLGDTEDSLVPQKIQAFEGIR.I
23	R.HTMALTSDGKLYGWGWNKFGQVGVGNNLDQCSPVQVR.F

Plants expressing GFP-UVR8 were grown for 10 days in white light (WL) and then exposed, or not, to 1.5 µmol m^{-2} s^{-1} UV-B for 24 h. The replicates for each treatment were obtained from 3 biologically independent experiments. GFP-UVR8 was immunoprecipitated from protein extracts and run on SDS-PAGE. Excised bands were subject to trypsin digestion prior to mass spectrometry (MS) analysis. MS files were analysed using Proteome Discoverer version 2.2.0.388 with Mascot as the search engine to identify peptides from the *Arabidopsis thaliana* Swiss-Prot database. Individual phosphopeptides are listed with Mascot ion scores; in this analysis ion scores above 33 exceed the 95% confidence threshold. Peptides with Mascot ion scores below 33 in each replicate are shaded in blue. Peptides were manually annotated to localise the site of phosphorylation using the Mascot delta score. Potential sites of phosphorylation are in red; those identified as phosphorylated are in bold. Where multiple sites are highlighted in a peptide, any of the sites could be phosphorylated.

Supplementary Table 3 | Measurement of phenolic compounds in different genotypes by HLPC.

	Genotype:	WT			S402A			S402D		
	Replicate:	1	2	3	1	2	3	1	2	3
**Flavonoid-glucoside**	**Retention time**	**Amount of phenolic compounds (mg/g dm)**								
Q-3-rut-7-rha	12.147	0.121	0.124	0.123	0.114	0.120	0.122	0.142	0.149	0.145
Q-3-diglc-7-rha	12.931	0.068	0.069	0.069	0.063	0.066	0.069	0.071	0.074	0.070
K-3-rut-7-rha	14.319	0.023	0.024	0.025	0.024	0.026	0.024	0.025	0.026	0.025
K-3-diglc-7-rha	15.899	0.064	0.065	0.061	0.058	0.065	0.063	0.093	0.097	0.097
Q-3-rha-7-glc	16.428	0.034	0.033	0.031	0.021	0.023	0.024	0.042	0.044	0.044
K-3-glc-7-rha	19.182	0.012	0.012	0.011	0.011	0.011	0.011	0.013	0.013	0.012
Q-3-rha-7-rha	20.212	0.164	0.164	0.160	0.144	0.149	0.156	0.183	0.191	0.187
K-3-rha-7-rha	24.222	0.006	0.006	0.006	0.006	0.005	0.006	0.006	0.006	0.006
	**Total:**	**0.491**	**0.497**	**0.486**	**0.441**	**0.465**	**0.475**	**0.575**	**0.598**	**0.586**
**HCAs**	**Retention time**									
Sinapoyl-glucoside	15.552	0.410	0.427	0.436	0.397	0.420	0.414	0.458	0.480	0.466
Sinapic acid	26.854	0.029	0.030	0.031	0.028	0.027	0.029	0.031	0.031	0.030
	**Total:**	**0.438**	**0.457**	**0.467**	**0.425**	**0.447**	**0.443**	**0.490**	**0.511**	**0.496**

GFP-UVR8 (WT), GFP-UVR8^{S402A} and GFP-UVR8^{S402D} plants were grown under a 16 h/8 h light/dark cycle in 100 µmol m^{-2} s^{-1} white light supplemented with 0.25 ±0.05 µmol m^{-2} s^{-1} narrowband UV-B for 14 days. The replicates for each genotype were obtained from 3 biologically independent experiments. Extracts of phenolic compounds were assayed for the presence of flavonoid-glucosides, and hydroxycinnamic acids (HCAs) by HPLC. Amounts are calculated per g of freeze-dried tissue (dm). Means ± S.E. are shown in Figure 4g. The flavonoid-glucosides detected were: quercitin-3-*O*-rutinoside-7-*O*-rhamnoside (Q-3-rut-7-rha); quercitin-3-*O*-diglucoside-7-*O*-rhamnoside (Q-3-diglc-7-rha); quercitin-3-*O*-rhamnoside-7-*O*-glucoside (Q-3-rha-7-glc); quercitin-3-*O*-rhamnoside-7-*O*-rhamnoside (Q-3-rha-7-rha); kaempferol-3-*O*-rutinoside-7-*O*-rhamnoside (K-3-rut-7-rha); kaempferol-3-*O*-diglucoside-7-*O*-rhamnoside (K-3-diglc-7-rha); kaempferol-3-*O*-glucoside-7-*O*-rhamnoside (K-3-glc-7-rha); kaempferol-3-*O*-rhamnoside-7-*O*-rhamnoside (K-3-rha-7-rha).

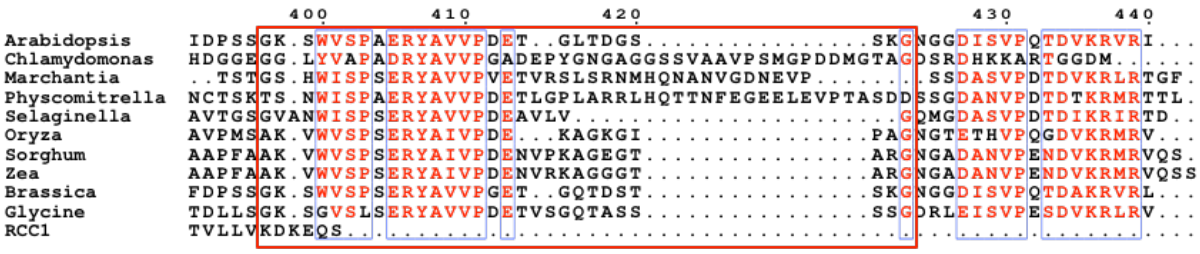

Supplementary Figure 1 | Conservation of S402 in UVR8 sequences from diverse taxa. Alignment of the last 48 amino acids of *Arabidopsis* UVR8 with plant orthologous proteins and human RCC1. The plant species shown are the single-cell green alga *Chlamydomonas reinhardtii*, bryophytes *Marchantia polymorpha* and *Physcomitrella patens*, lycophyte *Selaginella moellendorffii*, monocots *Oryza sativa (subspecies japonica)*, *Sorghum bicolor* and *Zea mays*, dicots *Brassica rapa* and *Glycine max*. Highly conserved amino acids are labelled in red. Highly conserved clusters are boxed in blue. The C27 region (amino acids 397-423) is boxed in red. The amino acid sequences of UVR8 from different species were obtained from UniProtKB or NCBI. The alignment was produced using MultAlin and the figure prepared using ESPript 3.0 [52].

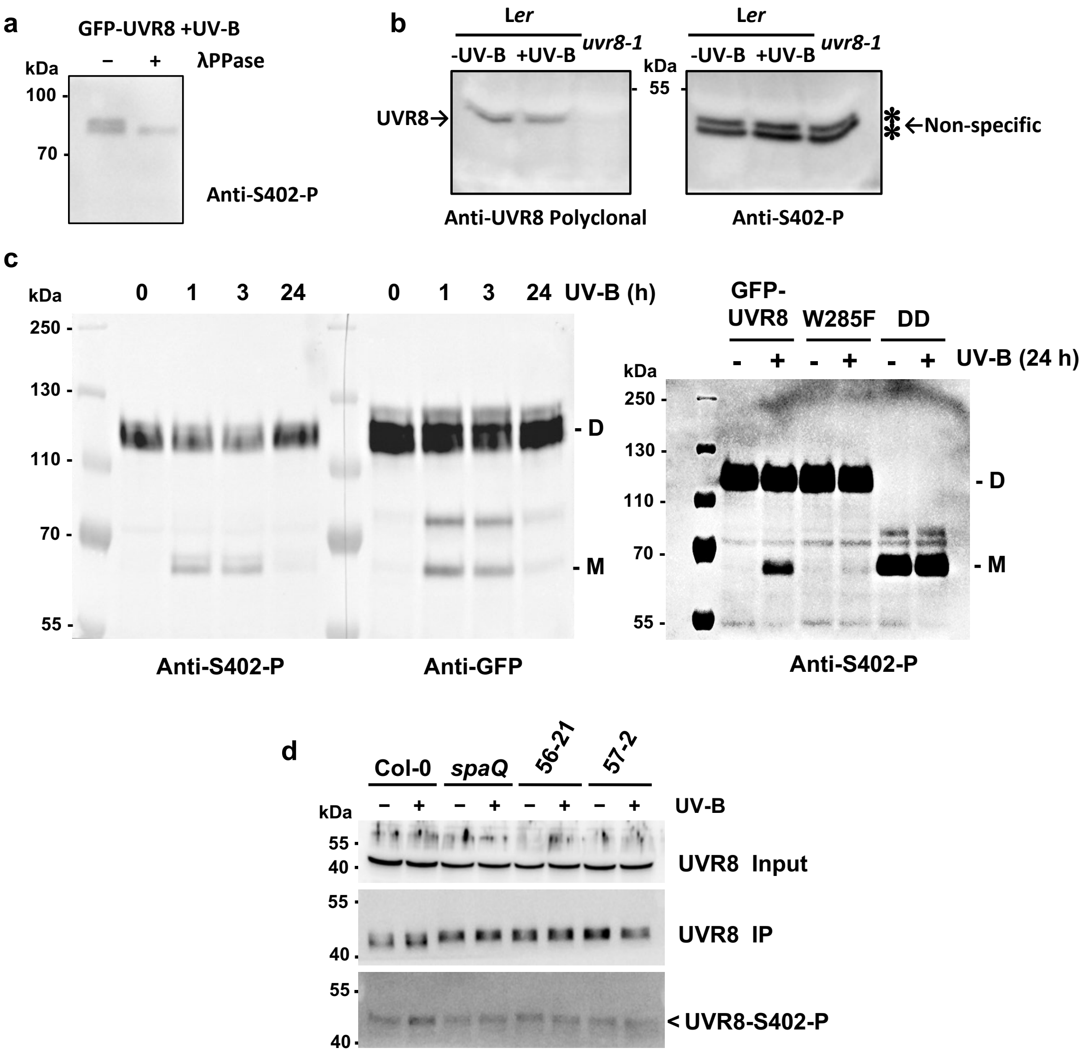

Supplementary Figure 2 | Characterisation of S402 phosphorylation. **a**, Protein extract from GFP-UVR8 plants was treated, or not, with lambda phosphatase. A western blot was probed with the anti-S402-P antibody. **b**, Western blot of protein extracts from L*er* and *uvr8-1* plants probed with either anti-UVR8 polyclonal antibody or anti-S402-P antibody. * identifies non-specific bands. **c**, Homozygous *uvr8-1* transgenic lines expressing either GFP-UVR8 or constitutively dimeric GFP-UVR8^{W285F} (W285F) or constitutively monomeric GFP-UVR8^{D96N,D107N} (DD) were exposed to UV-B as indicated. Protein extracts were prepared and SDS-treated non-boiled samples were run on SDS-PAGE. Left panel: duplicate western blots probed with either anti-GFP antibody to show the presence of dimer (D) and monomer (M) bands, or anti-S402-P antibody. Right panel: western blot probed with anti-S402-P antibody. **d**, 12-day old Col-0, *spaQ* or 2 different transgenic lines (56-21 and 57-2) of *spaQn* expressing SPA1^{R517E} were exposed to 3 µmol m^{-2} s^{-1} UV-B for 6 hours. Upper panel: UVR8 in protein extracts used for immunoprecipitation (Input); western blot probed with anti-UVR8 polyclonal antibody. Middle panel: UVR8 immunoprecipitates (IP); western blot probed with anti-UVR8 polyclonal antibody.

Lower panel: the IPs were probed with anti-UVR8$^{S402\text{-}P}$ phospho-antibody; the S402-P band is arrowed.
Representative blots are shown for n=3 biologically independent experiments, each of which gave similar results.

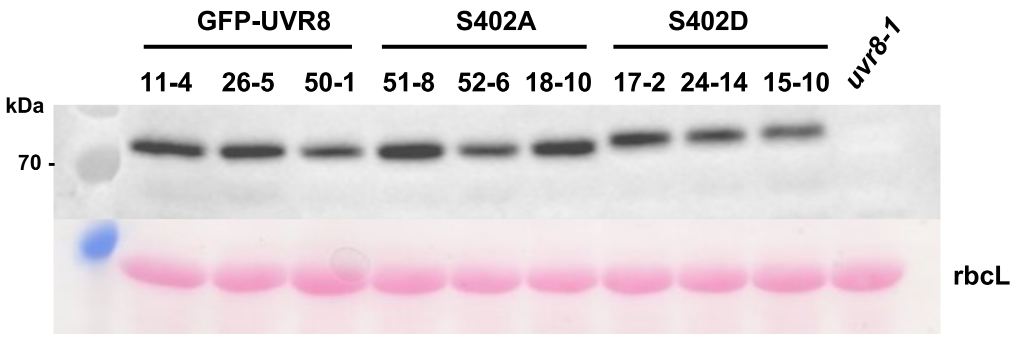

Supplementary Figure 3 | Expression in transgenic lines. Protein extracts were prepared from homozygous *uvr8-1* transgenic lines expressing either GFP-UVR8, GFP-UVR8^{S402A} (S402A) or GFP-UVR8^{S402D} (S402D). Three lines of each genotype are shown. A western blot was probed with anti-GFP antibody to show relative amounts of transgene expression. Ponceau-stained Rubisco large subunit (rbcL) is shown as a loading control.

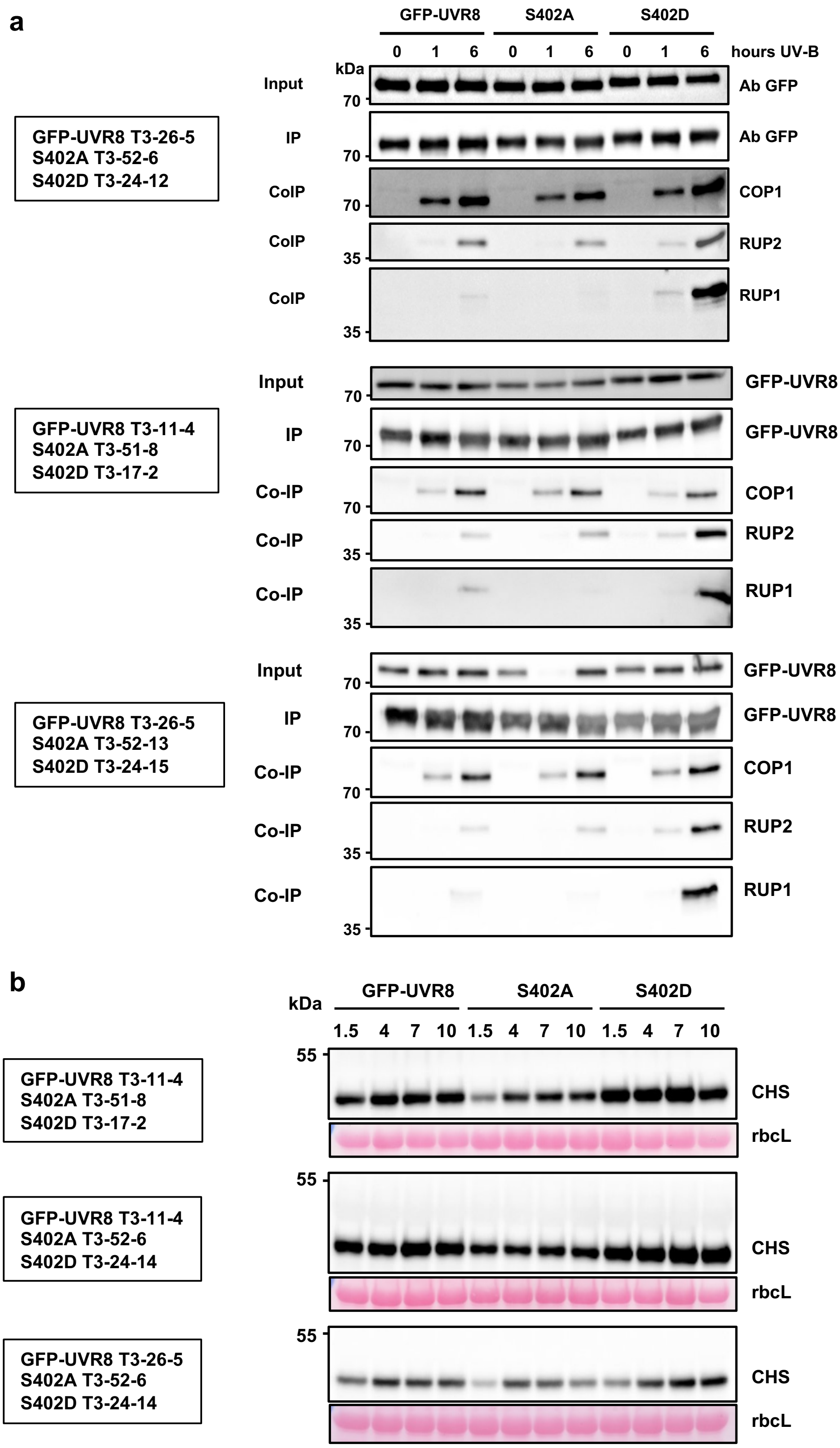

Supplementary Figure 4 | Reproducibility of data from multiple transgenic lines.
a, Co-immunoprecipitation assays showing interaction of GFP-UVR8, GFP-

UVR8^{S402A} (S402A) and GFP-UVR8^{S402D} (S402D) with COP1, RUP1 and RUP2 in 3 biologically independent experiments with different T3 transgenic lines, indicated to the left. Experimental conditions exactly as shown in Figure 3a. **b**, Assays of CHS accumulation in 3 biologically independent experiments with different T3 transgenic lines. Experimental conditions exactly as shown in Figure 4c.

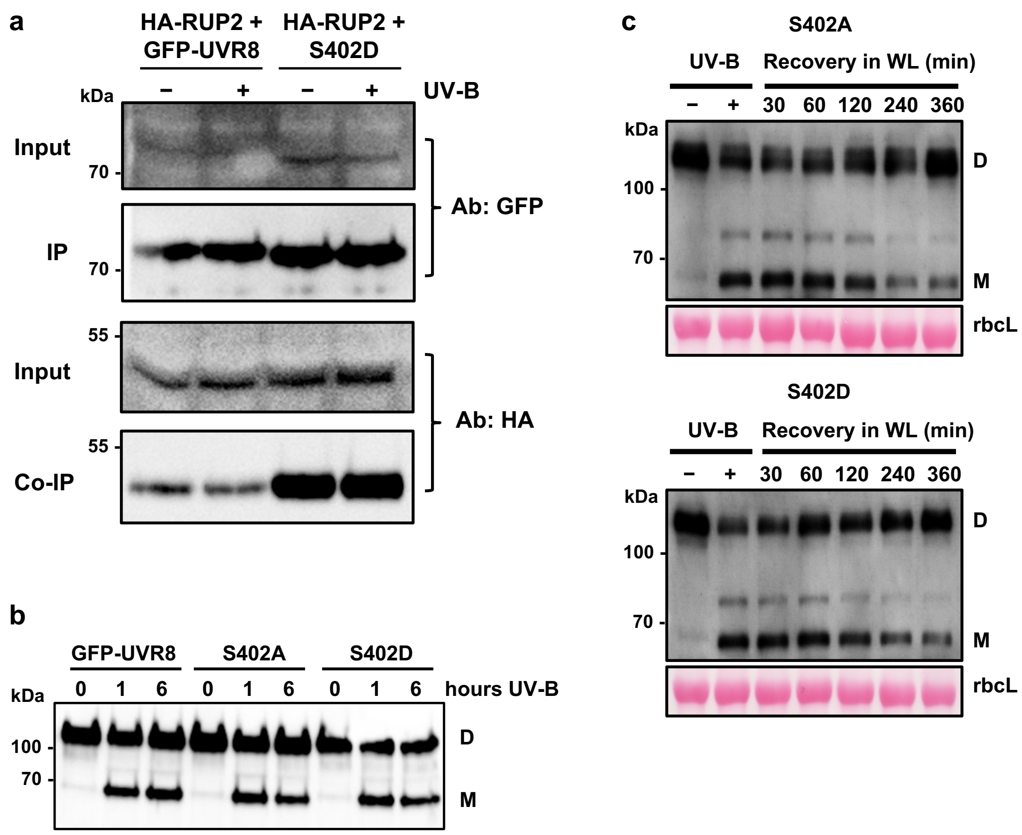

Supplementary Figure 5 | Effect of S402 phosphorylation on RUP interaction with UVR8. a, Enhanced interaction between UVR8^{S402D} and RUP2 in *Nicotiana* transient expression. *Nicotiana benthamiana* leaves were infiltrated with plasmids to express either GFP-UVR8 or GFP-UVR8^{S402D} (S402D) together with HA-RUP2. After 60 hours, leaves were exposed, or not, to 3 µmol m^{-2} s^{-1} UV-B for 3 hours. Protein extracts were run on SDS-PAGE and relative amounts of the expressed proteins were immunodetected on Western blots using anti-GFP or anti-HA antibodies (Ab) (Input). GFP-UVR8 or S402D were immunoprecipitated from the extracts and detected on western blots using anti-GFP antibody (IP); co-immunoprecipitated (Co-IP) RUP2 was detected using anti-HA antibodies. **b**, Dimer (D) and monomer (M) formation in GFP-UVR8, GFP-UVR8^{S402A} (S402A) and GFP-UVR8^{S402D} (S402D) plants either not exposed to UV-B or exposed to UV-B for 1 or 6 hours. Proteins were detected on a western blot using anti-GFP antibody. **c**, Kinetics of re-dimerisation in GFP-UVR8^{S402A} (S402A) and GFP-UVR8^{S402D} (S402D). Plants were grown in white light minus UV-B, exposed to UV-B to induce monomerisation, and returned to white light minus UV-B for the times indicated. UVR8 proteins were detected as in **b**. Ponceau-stained rbcL is shown as a loading control. Representative blots are shown for n=3 biologically independent experiments, each of which gave similar results.

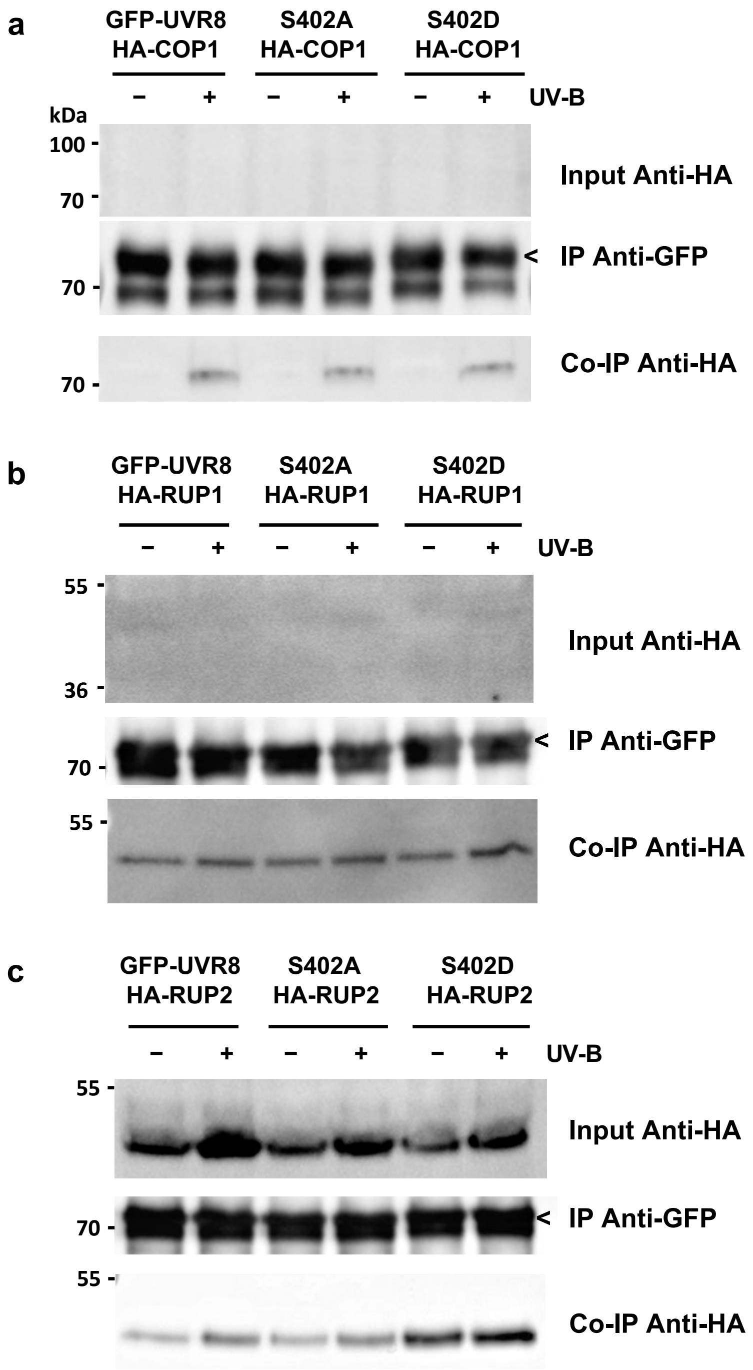

Supplementary Figure 6 | Interaction of UVR8 with proteins in mammalian cells. GFP-UVR8, GFP-UVR8^{S402A} (S402A) and GFP-UVR8^{S402D} (S402D) were co-expressed in mammalian cells with either **a**, HA-COP1, **b**, HA-RUP1, or **c**, HA-RUP2. Cells were exposed, or not, to UV-B. GFP-UVR8 fusions (arrowed) were immunoprecipitated from protein extracts and detected on western blots using anti-GFP antibody (IP). Co-immunoprecipitated (Co-IP) COP1, RUP1 or RUP2 were detected using anti-HA antibody. COP1 and RUP1 were below the limit of detection in total protein extracts (Input), whereas RUP2 expression was detected. Representative blots are shown for n=3 biologically independent experiments, each of which gave similar results.

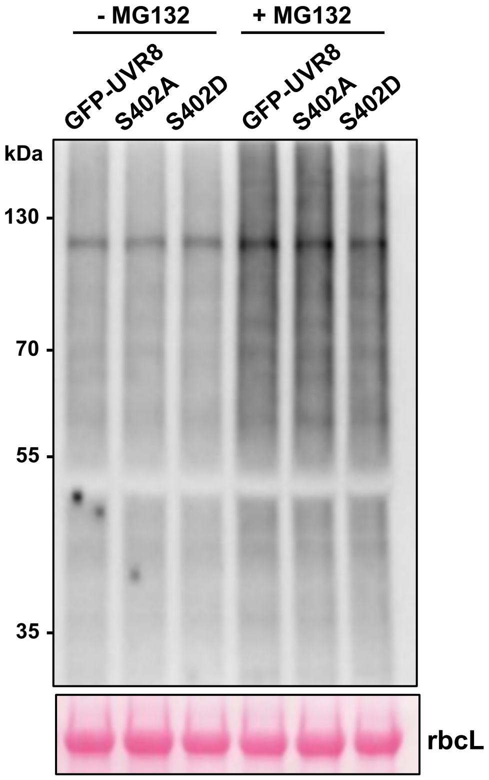

Supplementary Figure 7 | Effect of MG132 on protein ubiquitylation. Transgenic *uvr8-1* plants expressing either GFP-UVR8, GFP-UVR8^{S402A} (S402A) or GFP-UVR8^{S402D} (S402D) were incubated, or not, with 100 µM MG132 for 11 hours prior to the start of the light period (100 µmol m^{-2} s^{-1} white light supplemented with 0.25 µmol m^{-2} s^{-1} UV-B). Protein extracts were prepared from the plants 4 hours after the start of illumination and a western blot was probed with anti-ubiquitin antibody to show relative amounts of ubiquitylated proteins. Ponceau-stained Rubisco large subunit (rbcL) is shown as a loading control. The western blot shown is from the experiment presented in Figure 4e.

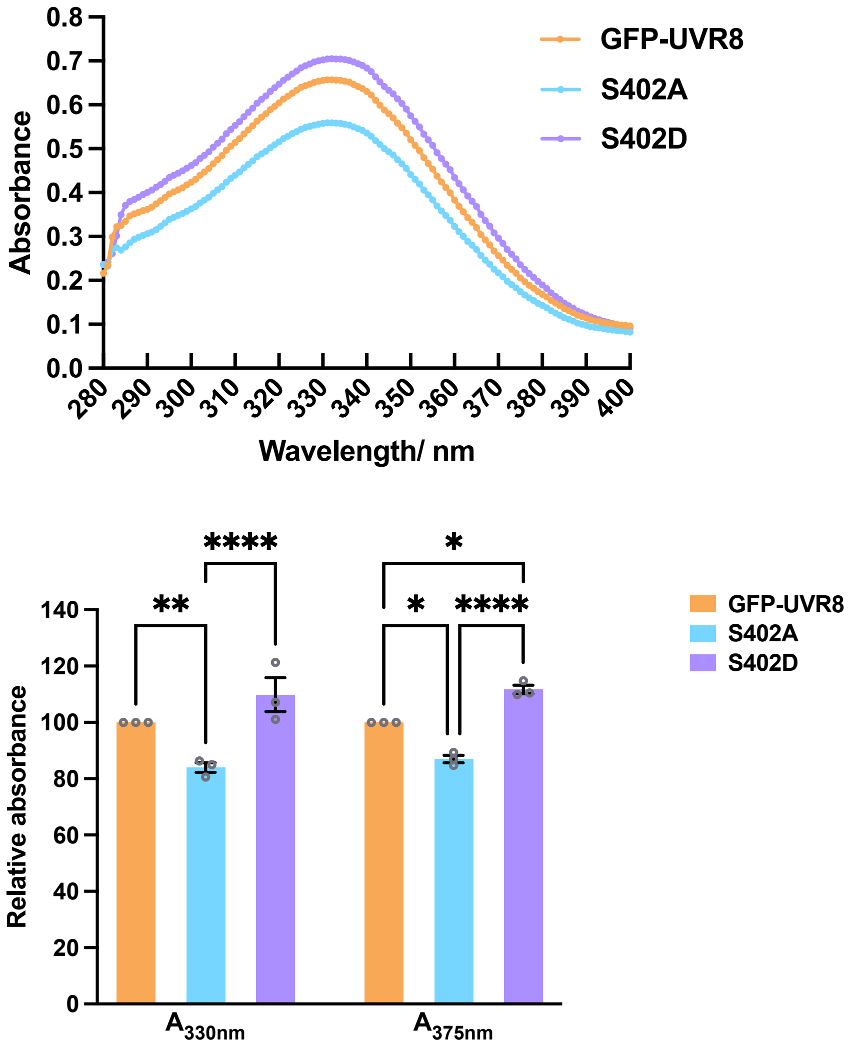

Supplementary Figure 8 | Relative amounts of UV-absorbing phenolic compounds in different lines. GFP-UVR8, GFP-UVR8^{S402A} (S402A) and GFP-UVR8^{S402D} (S402D) plants were grown under a 16 h/8 h light/dark cycle in 100 µmol m^{-2} s^{-1} white light supplemented with 0.25 ±0.05 µmol m^{-2} s^{-1} narrowband UV-B for 14 days. Absorbance spectra were measured for extracts of phenolic compounds. **a**, Spectra obtained in one experiment, representative of n=3 biologically independent experiments, each of which gave similar results. **b**, Mean absorbances at 330 nm (maximal absorbance of hydroxycinnamic acids) and 375 nm (maximal absorbance of flavonoids) normalised to GFP-UVR8 to facilitate comparison. Means values ± S.E. are shown for n=3 biologically independent experiments. The data were analysed using two-way ANOVA with Tukey's multiple comparisons test; for 330 nm, p values for the differences between GFP-UVR8 and S402D, GFP-UVR8 and S402A, and S402D and S402A are p=0.0560, p=0.0031 (**) and p<0.0001 (****) respectively; for 375 nm, p values for the differences between GFP-UVR8 and S402D, GFP-UVR8 and S402A, and S402D and S402A are p=0.0236 (*), p=0.0129 (*) and p<0.0001 (****) respectively.
