# Supplementary material for: Phosphorylation of Arabidopsis UVR8 photoreceptor modulates protein interactions and responses to UV-B radiation
Source: Nat Commun. 2024 Feb 9;15:1221. doi: 10.1038/s41467-024-45575-7 (PMC10858049; doi:10.1038/s41467-024-45575-7)
Supplement: Supplementary file 3 — Reporting Summary [file 41467_2024_45575_MOESM3_ESM.pdf]

nature portfolio

Corresponding author(s): Gareth Jenkins

Last updated by author(s): Jan 16, 2024

# Reporting Summary

Nature Portfolio wishes to improve the reproducibility of the work that we publish. This form provides structure for consistency and transparency in reporting. For further information on Nature Portfolio policies, see our Editorial Policies and the Editorial Policy Checklist.

## Statistics

For all statistical analyses, confirm that the following items are present in the figure legend, table legend, main text, or Methods section.

| n/a | Confirmed | |
|---|---|---|
| ☐ | ☒ | The exact sample size (*n*) for each experimental group/condition, given as a discrete number and unit of measurement |
| ☐ | ☒ | A statement on whether measurements were taken from distinct samples or whether the same sample was measured repeatedly |
| ☐ | ☒ | The statistical test(s) used AND whether they are one- or two-sided<br>*Only common tests should be described solely by name; describe more complex techniques in the Methods section.* |
| ☒ | ☐ | A description of all covariates tested |
| ☐ | ☒ | A description of any assumptions or corrections, such as tests of normality and adjustment for multiple comparisons |
| ☐ | ☒ | A full description of the statistical parameters including central tendency (e.g. means) or other basic estimates (e.g. regression coefficient) AND variation (e.g. standard deviation) or associated estimates of uncertainty (e.g. confidence intervals) |
| ☐ | ☒ | For null hypothesis testing, the test statistic (e.g. *F*, *t*, *r*) with confidence intervals, effect sizes, degrees of freedom and *P* value noted<br>*Give P values as exact values whenever suitable.* |
| ☒ | ☐ | For Bayesian analysis, information on the choice of priors and Markov chain Monte Carlo settings |
| ☒ | ☐ | For hierarchical and complex designs, identification of the appropriate level for tests and full reporting of outcomes |
| ☒ | ☐ | Estimates of effect sizes (e.g. Cohen's *d*, Pearson's *r*), indicating how they were calculated |

*Our web collection on statistics for biologists contains articles on many of the points above.*

## Software and code

Policy information about availability of computer code

| | |
|---|---|
| Data collection | A Thermo Fisher Scientific Q Exactive HF or Q Exactive Plus mass spectrometer interfaced with a 3000 RSLC Nano liquid chromatography system was used for mass spectrometry analysis of protein samples.<br>A StepOnePlus Real-Time PCR System (Applied Biosystems, Life Technologies) was used for qPCR assays of transcripts.<br>Phenolic compounds were analysed using a Shimadzu Prominence HPLC system (Shimadzu, Kyoto, Japan). |
| Data analysis | Raw mass spectrometry files were analysed using Proteome Discoverer version 2.2.0.388 with Mascot (Matrix Science) as the search engine.<br>ImageJ Version: 2.0.0-rc-65/1.51w (https://ImageJ.net) was used to quantify relative band intensities on western blots and autoradiographs.<br>Statistical analysis was performed with Microsoft Excel and GraphPad Prism 9 software. |

For manuscripts utilizing custom algorithms or software that are central to the research but not yet described in published literature, software must be made available to editors and reviewers. We strongly encourage code deposition in a community repository (e.g. GitHub). See the Nature Portfolio guidelines for submitting code & software for further information.

## Data

Policy information about availability of data

All manuscripts must include a data availability statement. This statement should provide the following information, where applicable:

- Accession codes, unique identifiers, or web links for publicly available datasets
- A description of any restrictions on data availability
- For clinical datasets or third party data, please ensure that the statement adheres to our policy

The data supporting the findings of this study are available within the paper and its Supplementary Information files (uncropped gel images and blots; source data for graphs). The mass spectrometry proteomics data have been deposited to the ProteomeXchange Consortium via the PRIDE partner repository with the dataset identifier PXD035649.

## Human research participants

Policy information about studies involving human research participants and Sex and Gender in Research.

| | |
|---|---|
| Reporting on sex and gender | N/A |
| Population characteristics | N/A |
| Recruitment | N/A |
| Ethics oversight | N/A |

Note that full information on the approval of the study protocol must also be provided in the manuscript.

# Field-specific reporting

Please select the one below that is the best fit for your research. If you are not sure, read the appropriate sections before making your selection.

[x] Life sciences [ ] Behavioural & social sciences [ ] Ecological, evolutionary & environmental sciences

For a reference copy of the document with all sections, see nature.com/documents/nr-reporting-summary-flat.pdf

# Life sciences study design

All studies must disclose on these points even when the disclosure is negative.

| | |
|---|---|
| Sample size | Sample sizes and results of statistical analysis are described in the Figure legends. Sample sizes were based on previous published studies with similar experiments and are sufficient to show whether differences between treatments were reproducible and statistically significant. |
| Data exclusions | No data was excluded from the analysis. |
| Replication | All the data are based on at least 3 independent biological replicates to ensure that results obtained were consistent, with the exception of the labeling experiment with mutants in Fig. 1b, which was undertaken in two independent experiments that gave very similar results. Information about replication is given in the Figure and Table legends. |
| Randomization | Sample allocation and selection was random. |
| Blinding | In most cases the same investigator performed the experimental treatment and the subsequent analysis, so blinding was not applicable. Analyses by collaborators in other laboratories were undertaken without prior knowledge of the plant material or treatments. |

# Reporting for specific materials, systems and methods

We require information from authors about some types of materials, experimental systems and methods used in many studies. Here, indicate whether each material, system or method listed is relevant to your study. If you are not sure if a list item applies to your research, read the appropriate section before selecting a response.

## Materials & experimental systems

| n/a | Involved in the study |
| --- | --- |
| ☐ | ☒ Antibodies |
| ☐ | ☒ Eukaryotic cell lines |
| ☒ | ☐ Palaeontology and archaeology |
| ☒ | ☐ Animals and other organisms |
| ☒ | ☐ Clinical data |
| ☒ | ☐ Dual use research of concern |

## Methods

| n/a | Involved in the study |
| --- | --- |
| ☒ | ☐ ChIP-seq |
| ☒ | ☐ Flow cytometry |
| ☒ | ☐ MRI-based neuroimaging |

# Antibodies

Antibodies used

Anti-GFP antibody (Chromotek, Germany) Cat. no. 3h9
Anti-GFP antibody (Clontech, Saint-Germain-en-Laye, France) Cat. no. 632375
Anti-HY5 antibody (Agrisera, Vännäs, Sweden) Cat. no. AS12 1867
Anti-ubiquitin antibody (Agrisera, Vännäs, Sweden) Cat. no. AS08 317
Anti-CHS antibody (Santa Cruz Biotechnology, Heidelberg, Germany) Cat. no. sc-12620
Anti-HA antibody (Roche, Basel, Switzerland) Cat. No. 3F10
Anti-GST antibody (GenScript, Oxford, UK) Cat. no. A00865
Anti-UVR8 polyclonal antibody (produced by the Jenkins laboratory: Soriano et al., reference 36),
Anti-COP1 antibody (obtained from Hong-Quan Yang; Lian et al., reference 37)
Anti-RUP1 and anti-RUP2 antibodies (produced by the Jenkins laboratory: Liao et al., reference 26)
Anti-UVR8S402-P phospho-antibody (produced by the Jenkins laboratory: this paper)

Validation

Commercially available antibodies were validated by the manufacturer and shown to detect the corresponding proteins on western blots of Arabidopsis proteins in this and other studies.
Anti-UVR8 polyclonal antibody was shown to recognize UVR8 by Soriano et al., reference 36.
Anti-COP1 antibody was characterized by Lian et al., reference 37.
Anti-RUP1 and anti-RUP2 antibodies were shown to detect the corresponding proteins by Liao et al., reference 26.
Anti-UVR8S402-P phospho-antibody is characterized in this paper.

# Eukaryotic cell lines

Policy information about cell lines and Sex and Gender in Research

Cell line source(s)

Human embryonic kidney (HEK) 293T

Authentication

Maintained in laboratories of Prof Graeme Milligan and Dr Brian Hudson at University of Glasgow for over 20 years and used in many published studies.

Mycoplasma contamination

N/A

Commonly misidentified lines
(See ICLAC register)

N/A
